# Comparison of percutaneous radiofrequency thermal ablation and surgical resection for small hepatocellular carcinoma

**DOI:** 10.1186/1471-230X-11-143

**Published:** 2011-12-28

**Authors:** Hiroki Nishikawa, Tadashi Inuzuka, Haruhiko Takeda, Jun Nakajima, Fumihiro Matsuda, Azusa Sakamoto, Shinichiro Henmi, Keiichi Hatamaru, Tetsuro Ishikawa, Sumio Saito, Akihiro Nasu, Ryuichi Kita, Toru Kimura, Akira Arimoto, Yukio Osaki

**Affiliations:** 1 Department of Gastroenterology and Hepatology, Osaka Red Cross Hospital, 5-30 Fudegasaki-cho, Tennoji-ku, Osaka, 543-0027, Japan; 2 Department of Surgery, Osaka Red Cross Hospital, 5-30 Fudegasaki-cho, Tennoji-ku, Osaka, 543-0027, Japan

**Keywords:** PRFA, surgical resection, HCC, survival, recurrence

## Abstract

**Background:**

The purpose of this investigation was to compare the outcome of percutaneous radiofrequency thermal ablation therapy (PRFA) with surgical resection (SR) in the treatment of single and small hepatocellular carcinoma (HCC).

**Methods:**

We conducted a retrospective cohort study on 231 treatment naive patients with a single HCC ≤ 3 cm who had received either curative PRFA (162 patients) or curative SR (69 patients). All patients were regularly followed up after treatment at our department with blood and radiologic tests.

**Results:**

The 1-, 3- and 5-year overall survival rates after PRFA and SR were 95.4%, 79.6% and 63.1%, respectively in the PRFA group and 100%, 81.4% and 74.6%, respectively in the SR group. The corresponding recurrence free survival rates at 1, 3 and 5 years after PRFA and SR were 82.0%, 38.3% and 18.0%, respectively in the PRFA group and 86.0%, 47.2% and 26.0%, respectively in the SR group. In terms of overall survival and recurrence free survival, there were no significant differences between these two groups. In comparison of PRFA group patients with liver cirrhosis (LC) (n = 127) and SR group patients with LC (n = 50) and in comparison of PRFA group patients without LC (n = 35) and SR group patients without LC (n = 19), there were also no significant differences between two groups in terms of overall survival and recurrence free survival. In the multivariate analysis of the risk factors contributing to overall survival, serum albumin level was the sole significant factor. In the multivariate analysis of the risk factors contributing to recurrence free survival, presence of LC was the sole significant factor. The rate of serious adverse events in the SR group was significantly higher than that in the PRFA group (P = 0.023). Hospitalization length in the SR group was significantly longer than in the PRFA group (P = 0.013).

**Conclusions:**

PRFA is as effective as SR in the treatment of single and small HCC, and is less invasive than SR. Therefore, PRFA could be a first choice for the treatment of single and small HCC.

## Background

Hepatocellular carcinoma is a major health problem worldwide, with an estimated incidence ranging between 500,000 and 1,000,000 new cases annually. It is the fifth most common cancer in the world and the third most common cause of cancer-related death [1]. The prognosis of HCC is generally poor. Surgical resection (SR) remains the best hope for a cure but is suitable for only 9 to 27% of patients [2,3]. The presence of significant background liver cirrhosis (LC) often precludes hepatic resection in patients with HCC. Recurrence in the liver remnant is also common in patients who have undergone radical hepatic resection.

Currently, local ablative therapy competes with surgical resection and liver transplantation as primary treatment for small HCC. Various locoregional therapies are used to treat patients who are not candidates for surgery because of the severity of the underlying liver disease. Percutaneous radiofrequency thermal ablation (PRFA), a recently developed local ablative technique, has attracted the greatest interest and popularity because of its efficacy and safety [4]. Previous studies have shown PRFA to give good results from the perspective of tumor control, with complete tumor ablation rates of 90 to 95%, and low local tumor progression rates of 5 to 10% [5-8]. Prospective randomized trials have shown PRFA to be better than percutaneous ethanol injection (PEI) in producing a higher rate of complete ablation with fewer numbers of treatment sessions [9]. However, there is still debate with regard to whether PRFA or SR is the most suitable therapy of small HCC.

In the present study, we conducted a retrospective cohort study to compare the results of PRFA and SR in the treatment of small HCC.

## Methods

### Patients and HCC diagnosis

Between January 2004 and January 2010, 231 patients with single HCC ≤ 3 cm in diameter received curative treatment using PRFA or SR in our department. Before performing PRFA or SR, a full discussion was made between physician and surgeon. After giving enough information including contents of the discussion between physician and surgeon to patients, patients themselves made decisions whether they received PRFA or SR. In patients with the tumor sites extremely difficult to perform PRFA such as the site directly under the hepatic dome or the heart or with poor visibility of the tumor under ultrasonography owing to extreme obesity or impossibility of breath hold when performing PRFA, SR was performed. And in patients whom high rates of complications were expected as when tumors at the site of hepatic hilar lesion were treated by PRFA, SR was performed. In patients whom informed consent could not be obtained upon SR for the reason such as physical burden, PRFA was performed. Even in patients with poor liver function such as Child-Pugh C, if they wished to treat HCC and there were no ascites, treatment for HCC was performed after fully explaining the risk for treatment. PRFA was administered to 162 patients and 69 patients underwent SR. Written informed consent was obtained from all patients. The ethics committee of our department approved the protocols for PRFA and SR. The present study comprised a retrospective analysis of patient records and all treatments were conducted in an open-label manner. The primary end point was overall survival and the secondary end point was recurrence free survival.

HCC was diagnosed using abdominal ultrasound and dynamic computed tomography (CT) scans (hyperattenuation during the arterial phase in all or some part of the tumor and hypoattenuation in the portal-venous phase) and/or magnetic resonance imaging (MRI), mainly based on the recommendations of the American Association for the Study of Liver Diseases [10]. Arterial and portal phase dynamic CT images were obtained at approximately 30 s and 120 s, respectively, after the injection of the contrast material. Abdominal angiography combined with CT (angio-CT) assistance was performed on all patients before PRFA and SR. This was due to the fact that Yamasaki et al. reported that this technique was useful for detecting small satellite nodules [11]. Then, we confirmed the presence of single HCC ≤ 3 cm in diameter with no vascular invasion using CT during hepatic arteriography (CTHA) and arterial-portography (CTAP). With regard to the diagnosis of liver cirrhosis, resected specimen at surgery was used in the SR group, and biopsy specimen was used in the PRFA group, respectively.

### PRFA procedure

We routinely used a cool-tip needle (Radionics Corp., Burlington, MA, USA) while performing PRFA. Using the intercostal or subcostal approach, a 17-gauge, 2 or 3 cm cooled-tip electrode was inserted under real-time ultrasound guidance. The initial treatment was planned with one ablation for tumors of < 2 cm in diameter, and two or more ablations with the overlapping technique for tumors of ≥ 2 cm in diameter. After insertion of the electrode into the tumor, we started ablation at 60 W for the 3-cm exposed tip and 40 W for the 2-cm exposed tip. The power was increased to 120 W at a rate of 10 W/min. The duration of a single ablation was 12 min for the 3-cm electrode and 6 min for the 2-cm electrode. After PRFA exposure, the pump was stopped and the temperature of the needle tip was measured. When the temperature reached > 60°C, additional ablation was not performed. When tumor ablation was complete, thermal ablation was performed along the needle track. All patients were carefully observed for treatment-related complications. All procedures were performed under ultrasound guidance by one of five operators who had at least 3 years of experience of performing PRFA. We used the artificial ascites technique to prevent collateral thermal injury when the anticipated PRFA zone was in contact with a critical organ, such as the hepatic flexure of the colon. We also used this technique to improve visibility when the index tumor was located in the hepatic dome area. In the present study, for all patients who had received PRFA, we confirmed that the ablative margin surrounded the entire circumference of the tumor by using dynamic 16-column multi-detector CT (MDCT) using 3-mm slice scans within 1 week after PRFA and 1 month after PRFA.

### Surgical resection procedure

All procedures were performed by one of four surgeons who had at least 10 years of experience of surgical resection. Surgical resection was carried out under general anesthesia using a right subcostal incision with a midline extension. We performed anatomic partial hepatectomy with a resection margin of at least 1 cm over the tumor, based on intraoperative ultrasonography (IOUS) guidance. IOUS was routinely performed to estimate the location, size, number and feeding vessels of the tumor, as well as to give an exact vascular map of liver anatomy. The Cavitron ultrasonic aspiration (CUSA, Valley Lab Corp, USA) was used to dissect the liver tissue. Hemostasis was achieved with dipolar electric coagulation and suturing. The Pringle maneuver was usually used in case of cirrhotic liver, with a clamp/unclamp time of 15 min/5 min policy. When liver function approached normal and adverse events had disappeared after surgical resection, we permitted patient discharge.

### Follow-up

Follow-up consisted of monthly blood tests and monitoring of tumor markers, including des-γ-carboxy prothrombin, which was measured using a chemiluminescent enzyme immunoassay (Lumipulse PIVKAII Eisai, Eisai, Tokyo, Japan). Dynamic CT scans were obtained every 3-4 months after PRFA and SR. No patients were lost to follow-up.

### Statistical analysis

Differences between the two groups were analyzed using the unpaired t-test for continuous variables, and the categorical variables were analyzed using the χ^2 ^test or continuity correction method. The overall survival curves and the recurrence-free survival curves were generated using the Kaplan-Meier method and compared using the log-rank test. The relative prognostic significance of the variables in predicting overall survival were assessed using univariate and multivariate Cox proportional hazards regression models. All variables with a P value < 0.05 evaluated using univariate analysis were subjected to multivariate analysis. Results of the multivariate analysis were presented as the hazard ratio (HR) with a corresponding 95% confidence interval (CI). All statistical tests were two-sided. All data were analyzed using SPSS software, version 9.0 (SPSS Inc., Chicago, IL, USA) for Microsoft Windows. Data are expressed as means ± standard deviation (SD). Values of P < 0.05 were considered to be statistically significant.

## Results

### Patient characteristics

The baseline characteristics of the two groups are shown in Table 1. Between the two groups, there were significant differences in tumor size (P = 0.001), platelet count (P = 0.004) and PIVKAII value (P = 0.037).

**Table 1:** Baseline characteristics of the percutaneous radiofrequency thermal ablation (PRFA) and surgical resection (SR) patient groups

	SR Group (n = 69)	PRFA Group (n = 162)	P value
Gender			
Male	50	95	0.054
Female	19	67	
Age	67.4 ± 9.7	68.4 ± 8.7	0.45
Tumor size (cm)	2.68 ± 0.49	1.9 9 ± 0.62	0.001
Etiology of liver disease			
chronic hepatitis B	8	9	0.182
chronic hepatitis C	51	135	
non B, non C	10	18	
Child-Pugh classification			
chronic hepatitis	19	35	0.285
Child-Pugh A	45	102	
Child-Pugh B	5	22	
Child-Pugh C	0	3	
Total-Bilirubin (mg/dL)	0.84 ± 0.48	0.93 ± 0.51	0.182
Serum albumin (g/dL)	3.89 ± 0.52	3.80 ± 0.55	0.248
Platelet ((×10^4^/mm^3^)	13.4 ± 6.3	11.2 ± 4.8	0.004
AST (IU/L)	58.9 ± 39.7	59.6 ± 34.0	0.897
ALT (IU/L)	53.0 ± 38.8	51.1 ± 32.7	0.697
Prothrombin time (%)	89.9 ± 14.9	87.8 ± 15.0	0.35
AFP (ng/mL)	376.7 ± 1989.8	74.7 ± 181.1	0.056
PIVKAII (mAU/mL)	739.0 ± 3900.1	95.5 ± 154.2	0.037

Data are expressed as the number of patients or the mean ± standard deviation. AST, aspartate aminotransferase; ALT, alanine aminotransferase; AFP, alpha-fetoprotein; PIVKAII, protein induced vitamin K absence or antagonist II

### SR group

Fifty-four of 69 patients were treated with segmentectomy; 12/69 patients received bisegmentectomy; 3/69 patients underwent hemihepatectomy. The histological diagnoses of 69 patients were as follows: well-differentiated hepatocellular carcinoma (3/69), moderately differentiated hepatocellular carcinoma (36/69) and poorly differentiated hepatocellular carcinoma (30/69). Using dynamic CT performed within 1 month after SR, we confirmed no residual HCC in the liver remnant of all patients.

### PRFA group

The mean number of treatment sessions for the 162 PRFA treated patients was 1.80 ± 0.37. Target biopsy prior to PRFA was not performed on any of the patients because of the specific complication of tumor seeding. We confirmed that all of the PRFA treated patients achieved complete ablation (ablated zone totally enveloped the tumor without enhancement) using dynamic CT prior to patient discharge and 1 month after PRFA. In the present study, there were 3 patients with Child-Pugh C who underwent PRFA. Their Child-Pugh scores were all 10 points and PRFA was performed safely in these 3 patients.

### Patient survival

The median follow-up period was 3.1 years (0.2-7 years) in the PRFA group and 3.3 years (0.7-7 years) in the SR group, respectively. Thirty-three patients (20.4%) in the PRFA group died during the follow-up period. The causes of death were HCC recurrence (24 patients), liver failure (6 patients) and miscellaneous (3 patients). Twelve patients (17.4%) in the SR group died during the follow-up period. The causes of death were HCC recurrence (9 patients), liver failure (2 patients) and miscellaneous (1 patient).

**Figure 1:**
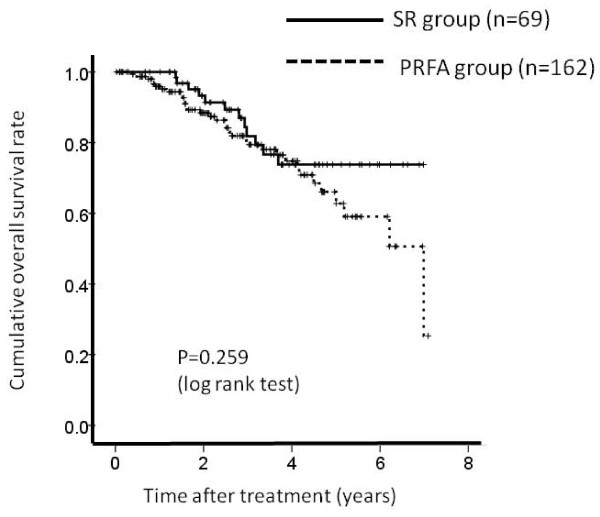
**Cumulative overall survival rate**. The 1-, 3- and 5-year overall survival rates after percutaneous radiofrequency thermal ablation (PRFA) and surgical resection (SR) were 95.4%, 79.6% and 63.1%, respectively in the PRFA group and 100%, 81.4% and 74.6%, respectively in the SR group. There was no significant difference between these two groups as determined using the log-rank test (P = 0.259).

**Figure 2:**
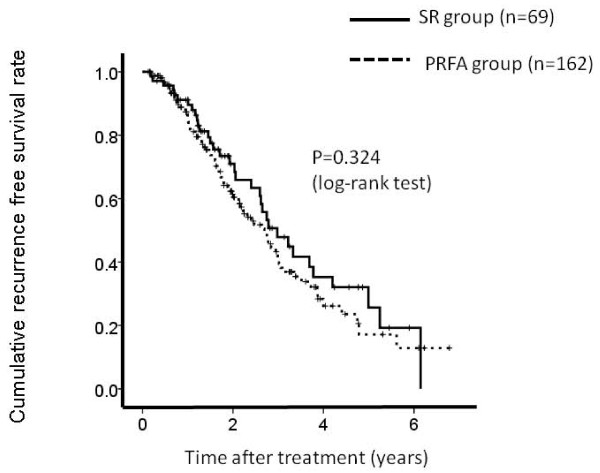
**Cumulative recurrence free survival rate**. The 1-, 3- and 5-year recurrence free survival rates after percutaneous radiofrequency thermal ablation (PRFA) and surgical resection (SR) were 82.0%, 38.3% and 18.0%, respectively in the PRFA group and 86.0%, 47.2% and 26.0%, respectively in the SR group There was no significant differences between these two groups as determined using the log-rank test (P = 0.324).

The 1-, 3- and 5-year overall survival rates after PRFA and SR were 95.4%, 79.6% and 63.1%, respectively in the PRFA group and 100%, 81.4% and 74.6%, respectively in the SR group (Figure 1). The corresponding recurrence free survival rates at 1, 3 and 5 years after PRFA and SR were 82.0%, 38.3% and 18.0%, respectively in the PRFA group and 86.0%, 47.2% and 26.0%, respectively in the SR group (Figure 2).

In terms of overall survival (P = 0.259) and recurrence free survival (P = 0.324), there were no significant differences between these two groups.

### Local tumor progression

**Figure 3:**
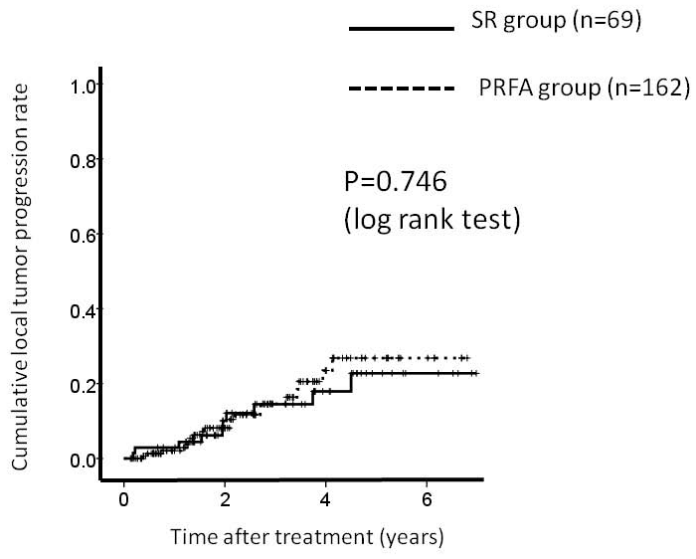
**Cumulative local tumor progression rate**. The 1-, 3- and 5-year local tumor progression rates after percutaneous radiofrequency thermal ablation (PRFA) and surgical resection (SR) were 2.0%, 14.3% and 28.3%, respectively in the PRFA group and 2.8%, 14.3% and 22.8%, respectively in the SR group. There was no significant differences between these two groups as determined using the log-rank test (P = 0.746).

We defined local tumor progression as the presence of a hypervascular nodule adjacent to the ablated area of PRFA or the resected area of SR using dynamic CT scan. 20 patients in the PRFA group and 10 patients in the SR group had local tumor progression during the observation period. The 1-, 3- and 5-year local tumor progression rates after PRFA and SR were 2.0%, 14.3% and 28.3%, respectively in the PRFA group and 2.8%, 14.3% and 22.8%, respectively in the SR group. (Figure 3) In terms of local tumor progression, there was no significant difference between these two groups (P = 0.746).

### Comparison between PRFA group patients with LC and SR group patients with LC

**Figure 4:**
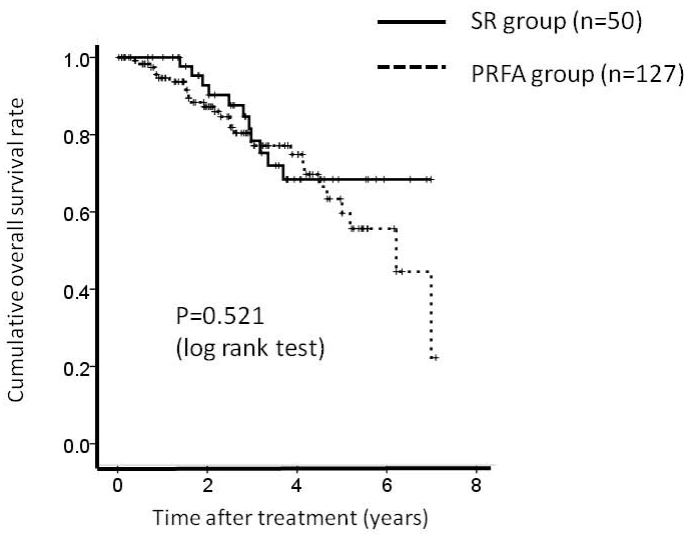
**Cumulative overall survival rate between percutaneous radiofrequency thermal ablation (PRFA) group patients with liver cirrhosis (LC) (n = 127) and surgical resection (SR) group patients with liver cirrhosis (LC) (n = 50)**. The 1-, 3- and 5-year overall survival rates after PRFA and SR were 94.2%, 75.8% and 56.4%, respectively in the PRFA group with LC and 100%, 78.0% and 67.8%, respectively in the SR group with LC. There was no significant differences between these two groups as determined using the log-rank test (P = 0.521).

**Figure 5:**
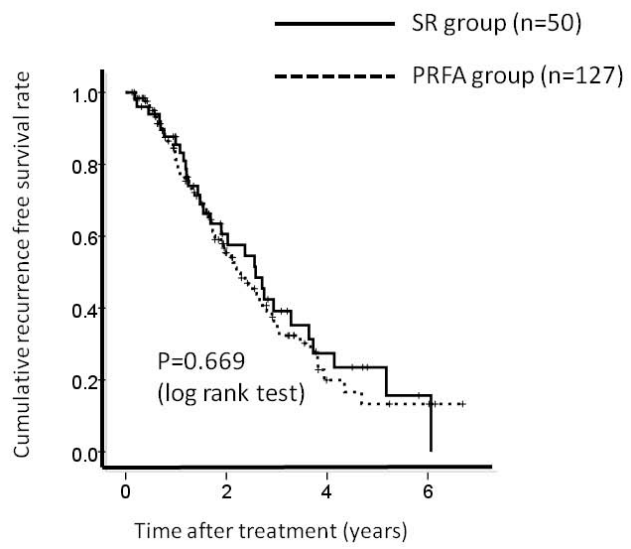
**Cumulative recurrence free survival rate between percutaneous radiofrequency thermal ablation (PRFA) group patients with liver cirrhosis (LC) (n = 127) and surgical resection (SR) group patients with liver cirrhosis (LC) (n = 50)**. The 1-, 3- and 5-year recurrence free survival rates after PRFA and SR were 86.0%, 35.0% and 14.8%, respectively in the PRFA group with LC and 79.5%, 39.3% and 23.8%, respectively in the SR group with LC. There was no significant differences between these two groups as determined using the log-rank test (P = 0.669).

There were 127 patients in PRFA group patients with LC and 50 patients in SR group patients with LC, respectively. The 1-, 3- and 5-year overall survival rates after PRFA and SR were 94.2%, 75.8% and 56.4%, respectively in the PRFA group with LC and 100%, 78.0% and 67.8%, respectively in the SR group with LC. (Figure 4) The corresponding recurrence free survival rates at 1, 3 and 5 years after PRFA and SR were 86.0%, 35.0% and 14.8%, respectively in the PRFA group with LC and 79.5%, 39.3% and 23.8%, respectively in the SR group with LC. (Figure 5) In terms of overall survival (P = 0.521) and recurrence free survival (P = 0.669), there were no significant differences between these two groups.

### Comparison between PRFA group patients without LC and SR group patients without LC

**Figure 6:**
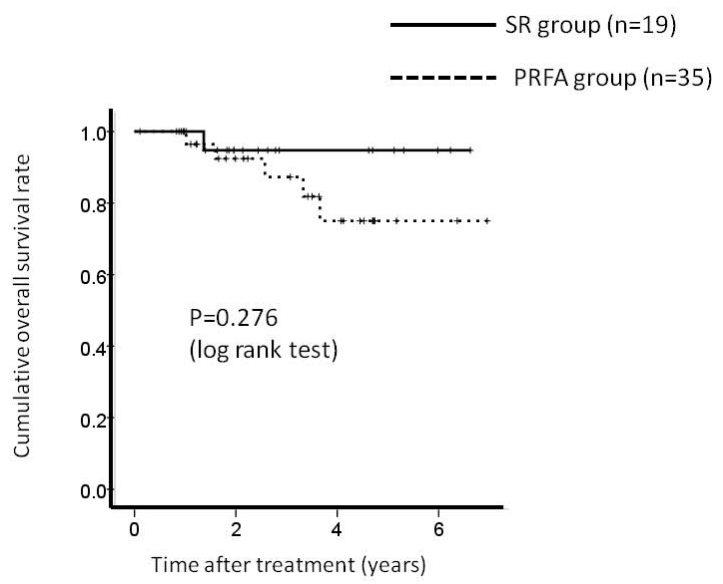
**Cumulative overall survival rate between percutaneous radiofrequency thermal ablation (PRFA) group patients without liver cirrhosis (LC) (n = 35) and surgical resection (SR) group patients without liver cirrhosis (LC) (n = 19)**. The 1-, 3- and 5-year overall survival rates after PRFA and SR were 96.6%, 87.2% and 74.4%, respectively in the PRFA group without LC and 100%, 95.6% and 95.6%, respectively in the SR group without LC. There was no significant differences between these two groups as determined using the log-rank test (P = 0.276).

**Figure 7:**
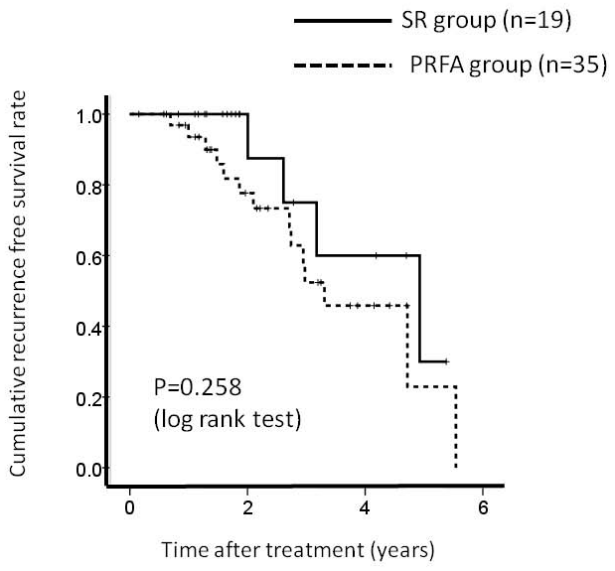
**Cumulative recurrence free survival rate between percutaneous radiofrequency thermal ablation (PRFA) group patients without liver cirrhosis (LC) (n = 35) and surgical resection (SR) group patients without liver cirrhosis (LC) (n = 19)**. The 1-, 3- and 5-year recurrence free survival rates after PRFA and SR were 93.0%, 52.5% and 22.2%, respectively in the PRFA group without LC and 100%, 75.7% and 30.4%, respectively in the SR group without LC. There was no significant differences between these two groups as determined using the log-rank test (P = 0.258).

There were 35 patients in PRFA group patients without LC and 19 patients in SR group patients without LC, respectively. The 1-, 3- and 5-year overall survival rates after PRFA and SR were 96.6%, 87.2% and 74.4%, respectively in the PRFA group without LC and 100%, 95.6% and 95.6%, respectively in the SR group without LC. (Figure 6) The corresponding recurrence free survival rates at 1, 3 and 5 years after PRFA and SR were 93.0%, 52.5% and 22.2%, respectively in the PRFA group with LC and 100%, 75.7% and 30.4%, respectively in the SR group with LC. (Figure 7) In terms of overall survival (P = 0.276) and recurrence free survival (P = 0.258), there were no significant differences between these two groups.

### Serious adverse events, hospitalization length and mortality

Serious adverse events were significantly more frequent in the SR group than in the PRFA group (6/69 versus 3/162; P = 0.023). Serious adverse events in the SR group were as follows: bile leakage (2 patients); refractory ascites (2 patients); acute respiratory distress syndrome (ARDS) (1 patient); and massive gastrointestinal bleeding (1 patient). Serious adverse events in the PRFA group were as follows: biloma (1 patient); refractory ascites (1 patient); and intra-abdominal bleeding (1 patient).

The hospitalization length was significantly longer in the SR group (18.1 ± 10.4 days) than in the PRFA group (14.7 ± 5.7 days) (P = 0.013). In addition, there was no patient who died within the same hospitalization, making the mortality rate 0% in two groups.

### Univariate and multivariate analysis of prognostic factors contributing to overall survival and recurrence free survival

In the univariate analysis of factors contributing to overall survival, hepatitis C virus (HCV) versus non HCV (P = 0.042), serum albumin (g/dL) (> 3.5 versus ≤ 3.5) (P = 0.003), and platelet count (× 10^4^/mm^3^) (> 10 versus ≤ 10) (P = 0.045) were found to be significant factors (Table 2). However, in the multivariate analyses involving these three factors, serum albumin (g/dL) (> 3.5 versus ≤ 3.5) was the sole significant factor contributing to overall survival.

**Table 2:** Univariate and multivariate analysis of the prognostic factors contributing to overall survival

		Multivariate Analysis	
		
Variables	Univariate Analysis	Hazard Ratio (95%CI)	P value
Surgical resection vs PRFA	0.257	0.964(0.870-1.224)	0.889
Gender (male vs female)	0.811		
Age (> 65 vs ≤ 65)	0.639		
HBV vs non HBV	0.074		
HCV vs non HCV	0.042	0.516 (0.163-1.628)	0.259
LC vs non LC	0.073		
Albumin (g/dL) (> 3.5 vs ≤ 3.5)	0.003	1.732 (1.015-3.314)	0.047
T-Bil (mg/dL) (> 1.0 vs ≤ 1.0)	0.141		
Platelet (×10^4^/mm^3^) (> 10 vs ≤ 10)	0.045	1.132 (0.568-2.258)	0.724
Prothrombin time (%) (> 80 vs ≤ 80)	0.068		
Serum AFP (> 100 vs ≤ 100)	0.987		
PIVKAII (> 100 vs ≤ 100)	0.15		
Tumor size (cm) (> 2 vs ≤ 2)	0.883		

PRFA, percutaneous radiofrequency thermal ablation; HBV, hepatitis B virus; HCV, hepatitis C virus; LC, liver cirrhosis; T-Bil, total bilirubin; CI, confidence interval; AFP, alpha-fetoprotein; PIVKAII, protein induced vitamin K absence or antagonist II

Similarly, in the univariate analysis of factors contributing to recurrence free survival, HCV versus non HCV (P = 0.022), LC versus non LC (P = 0.002) and platelet count (× 10^4^/mm^3^) (> 10 versus ≤ 10) (P = 0.005) were found to be significant factors (Table 3). However, in the multivariate analyses involving these three factors, the presence of LC was the sole significant factor contributing to recurrence free survival.

**Table 3:** Univariate and multivariate analysis of the prognostic factors contributing to recurrence free survival

		Multivariate Analysis	
		
Variables	Univariate Analysis (P value)	Hazard Ratio (95%CI)	P value
Surgical resection vs PRFA	0.324	0.988(0.896-1.124)	0.902
Gender (male vs female)	0.605		
Age (> 65 vs ≤ 65)	0.392		
HBV vs non HBV	0.399		
HCV vs non HCV	0.022	0.630 (0.367-1.079)	0.093
LC vs non LC	0.002	0.586 (0.334-1.027)	0.048
Albumin (g/dL) (> 3.5 vs ≤ 3.5)	0.531		
T-Bil (mg/dL) (> 1.0 vs ≤ 1.0)	0.703		
Platelet (×10^4^/mm^3^) (> 10 vs ≤ 10)	0.005	1.334 (0.889-2.001)	0.125
Prothrombin time (%) (> 80 vs ≤ 80)	0.696		
Serum AFP (> 100 vs ≤ 100)	0.857		
PIVKAII (> 100 vs ≤ 100)	0.807		
Tumor size (cm) (> 2 vs ≤ 2)	0.389		

PRFA, percutaneous radiofrequency thermal ablation; HBV, hepatitis B virus; HCV, hepatitis C virus; LC, liver cirrhosis; T-Bil, total bilirubin; CI, confidence interval; AFP, alpha-fetoprotein; PIVKAII, protein induced vitamin K absence or antagonist II

## Discussion

Partial hepatectomy in patients with resectable HCC, who have normal liver function and are in good general condition is still considered the gold standard therapy with the aim of delivering curability [12]. In recent years, it has been possible to reduce perioperative mortality to less than 5% depending on the extent of resection and hepatic reserve [13]. The improved outcome is primarily as a result of advances in surgical and radiologic techniques, perioperative care and more cautious patient selection [14].

Patients not eligible for resection because of their medical condition might be candidates for local ablative therapy, such as percutaneous ethanol injection (PEI) and PRFA. Many clinical trials comparing PRFA and PEI have demonstrated the clear superiority of PRFA over PEI [9,15-17]. However, a major limitation of PRFA is the small volume of tumor that can be treated. The rate of complete ablative necrosis decreases with the size of the tumor, particularly in the case of tumors larger than 3 cm. There is general consensus that complete response to PRFA therapy in patients is associated with improved outcome [18-20]. Therefore, in the present study, objectives were limited to patients with HCC ≤ 3 cm in size.

HCC mainly disseminates through the portal and hepatic veins. The micro-dissemination can invade the tributaries of the portal branches and shed tumor emboli in the neighboring branches of the same liver segment [21-24]. However, in the present study, with regard to recurrence free survival, there was no significant difference between the two treatment groups. One possible reason for this is that a sufficient ablative margin around the tumor when PRFA is administered may suppress the invasion of the micro-dissemination. Previous studies have reported that the initial treatment contributes to the survival of HCC patients treated using PRFA [19,25]. In PRFA therapy, obtaining sufficient ablative margin around the tumor seems to be essential.

The findings of the present study indicated that the overall and recurrence free survivals were the same for patients with a single HCC ≤ 3 cm in diameter treated with either PRFA or SR. In addition, PRFA was demonstrated to have an advantage over SR in causing less serious adverse events and a shorter hospitalization length. Chen et al conducted a randomized control trial (RCT) on 180 patients with a single HCC ≤ 5 cm to receive either PRFA or surgical resection [12], and Lu et al carried out another RCT on 105 patients with early HCC [26]. These two RCTs presented similar findings to those of our study. Additionally, four non-randomized controlled studies also reported similar findings of ours [27-30]. And our study suggests that PRFA is less invasive than SR. It seems that PRFA can be a first choice for the treatment of small HCC. On the other hand, a recent study indicated that surgical resection provided better survival and lower recurrence rates than RFA for patients with HCC that conformed to the Milan criteria for a RCT [31]. However, in comparing their results with ours, the mean age of their patient population was more than 10 years younger than ours. In the etiology of liver disease in their study, patients with chronic hepatitis B were in the majority [31]. However, in our study, patients with chronic hepatitis C were in the majority. Therefore, their study results did not reflect the actual situation in Japan where Japanese HCC patients consist of many elderly patients, and the etiology of background liver disease involves chronic hepatitis C which accounts for about 80% of Japanese HCC patients. Hence, we should interpret their study results with caution.

Our study had several limitations. First, it was a retrospective cohort study. Patients who had a good hepatic reserve tended to receive surgical resection, and this could have possibly led to bias. Second, we did not assess the histopathologic diagnosis of HCC in the PRFA group. Tateishi et al reported that patients with poorly differentiated HCC had a poorer outcome than patients with well to moderately differentiated HCC after PRFA [32]. Third, our study patients were limited to patients who have undergone curative treatment. These problems should be resolved in a future prospective study.

## Conclusion

In conclusion, we demonstrated that PRFA is as effective as SR in the treatment of single and small HCC patients who have undergone curative treatment, and that PRFA is less invasive than SR. Therefore, PRFA can be a first choice for the treatment of single and small HCC.

## Competing interests

The authors declare that they have no competing interests. In addition, none of the authors had any financial relationship (within the past 12 months) with a biotechnology manufacturer, a pharmaceutical company, or other commercial entity that has any interest in the subject matter, materials, or processes discussed in the manuscript.

## Authors' contributions

YO participated in the design of the study and performed the statistical analysis, and all authors read and approved the final manuscript.

## Pre-publication history

The pre-publication history for this paper can be accessed here:

http://www.biomedcentral.com/1471-230X/11/143/prepub
